# The Zinc-Dependent Protease Activity of the Botulinum Neurotoxins

**DOI:** 10.3390/toxins2050978

**Published:** 2010-05-07

**Authors:** Frank J. Lebeda, Regina Z. Cer, Uma Mudunuri, Robert Stephens, Bal Ram Singh, Michael Adler

**Affiliations:** 1 US Army Medical Research and Materiel Command, Ft. Detrick, MD 21702-5012, USA; 2 Bioinformatics Support Group, Advanced Biomedical Computing Center, Information Systems Program, SAIC-Frederick Inc., NCI-Frederick, Frederick, MD 21702, USA; Email: cerr@mail.nih.gov (R.Z.C.); (U.M.); (R.S.); 3 Botulinum Research Center, University of Massachusetts Dartmouth, 285 Old Westport Road, Dartmouth, MA 02747, USA; Email: bsingh@umassd.edu (B.R.S.); 4 US Army Medical Research Institute of Chemical Defense, Aberdeen Proving Ground, MD 21010-5400, USA; Email: michael.adler@us.army.mil (M.A.)

**Keywords:** catalysis, energy, k_cat_, K_m_, superactivation

## Abstract

The botulinum neurotoxins (BoNT, serotypes A-G) are some of the most toxic proteins known and are the causative agents of botulism. Following exposure, the neurotoxin binds and enters peripheral cholinergic nerve endings and specifically and selectively cleaves one or more SNARE proteins to produce flaccid paralysis. This review centers on the kinetics of the Zn-dependent proteolytic activities of these neurotoxins, and briefly describes inhibitors, activators and factors underlying persistence of toxin action. Some of the structural, enzymatic and inhibitor data that are discussed here are available at the botulinum neurotoxin resource, BotDB (http://botdb.abcc.ncifcrf.gov).

## 1. Introduction

This review focuses on the enzymatic function, thermodynamic properties and susceptibility to inhibitors and activators of the botulinum neurotoxins (BoNTs), the causative agents of botulism. These neurotoxins are metalloproteases (E.C. 3.4.24.69) that belong to the gluzincin clan [1] and the MA(E) clan/M27 family as classified by the MEROPS database [2]. These multi-domain bacterial proteins are produced by *Clostridium botulinum* and related species (*C. argentinense, baratii, butyricum*) and are categorized into seven immunologically distinct serotypes (A-G). In cases of food poisoning, the ingested toxin migrates from the gastrointestinal tract and eventually reaches its primary targets, the peripheral cholinergic nerve terminals to cause flaccid paralysis. The botulinum neurotoxins (BoNTs) are released from the bacteria in complex with various non-toxic proteins that serve to protect the neurotoxin from the degrading proteolytic and low pH environments found in the gastrointestinal tract [3]. 

An eighth homologous neurotoxin produced by *C. tetani* (tetanus neurotoxin, TeNT) causes spastic paralysis (tetanus) and is unaccompanied by accessory proteins [3]. Interestingly, TeNT and BoNT/B share the same molecular target (VAMP) and specifically cleave at the same peptide bond (see Section 2). In contrast to the BoNTs, tetanus neurotoxin (TeNT) enters these same cholinergic termini, is retrogradely transported within motor nerve axons to the spinal cord and is translocated into inhibitory neurons where it produces disinhibition leading to spastic paralysis [4,5]. Thus, the same general mechanism of proteolytic action produces two distinct symptoms that are dependent on their cellular location [6]. Moreover, at concentrations higher than those encountered *in vivo*, TeNT produces flaccid paralysis in isolated nerve-muscle preparations [5]. 

Clinical signs and symptoms of botulism consist of an assortment of abnormalities including those related to the neuromuscular junction and the autonomic nervous systems: dilated pupils, descending symmetric flaccid paralysis, dizziness, blurred vision, dry mouth, sore throat, constipation, nausea, vomiting, abdominal cramps, diarrhea, and paresthesia [7]. 

From a basic research perspective, these homologous neurotoxins have been exploited as pharmacologic tools for elucidating the role of the conserved SNAREs that regulate neurally evoked, Ca^2+^-dependent release of neurotransmitter from synaptic vesicles. In stark contrast to their roles as poisons, the type A and B toxins have been commercially developed as therapeutic agents for a variety of focal dystonias, autonomic dysfunctions, and cosmetic treatments [8,9,10]. 

**Figure 1:**
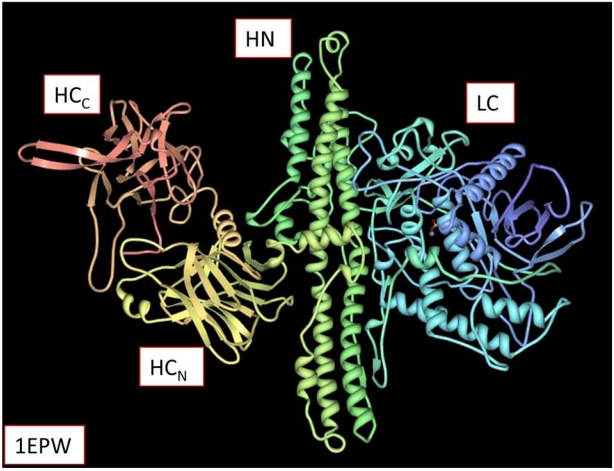
Representation of the four structural domains of the BoNT/B holotoxin (PDB ID 1EPW) [16]. The “Structures” section of the BotDB presently contains 90 3D protein structures found in the Protein Data Bank (PDB) [5]. From left to right, the order of these domains is: HC_C_, (red) HC_N_, (yellow-orange) HN (green), LC (blue).

These bacterial neurotoxins (molecular weight ~150 kDa) have four domains that comprise their three-dimensional (3D) structures (Figure 1) [11,12,13,14,15]. BoNT/A was the first holotoxin serotype to be resolved crystallographically [17] (PDB ID: 3BTA). The zinc-dependent proteolytic activities of the light chains (LCs) of BoNT serotypes A, B, and E were first reported in 1992 by Schiavo *et al.* [18] and the metalloprotease activity for the structurally homologous TeNT light chain was published during the same year [19]. When expressed, the neurotoxin molecule (progenitor toxin) is a single polypeptide chain. An initial post-translational modification is ‘nicking’, in which several amino acid residues are removed about a third of the way downstream from the *N*-terminus. This modification results in two chains that are connected by a disulfide bridge. 

The *N*-terminal domain of BoNT, the LC (~50 kDa), is displayed on the right-hand side in Figure 1 and functions as a zinc-dependent protease. The SCOP database [20] classifies these LCs as a mixture comprised of a-helices + b-strands, remarkably similar to the metalloprotease domain of anthrax lethal factor [21]. The LC is widely accepted to be the toxic moiety of the BoNTs. Although much is known about the role of the BoNT domains, there is no explanation available as to the mechanism involved in the separation of the heavy and light chains that are tightly held together by ionic interactions [22]. Under laboratory conditions this separation of chains typically occurs in a high pH, reducing, high ionic strength environment, *i.e.*, pH 8.4 buffer containing 0.01 M dithiothreitol, 2 M urea, and 0.2 M NaCl [23]. Further studies are required to characterize the mechanism of this separation as it is assumed to occur in living cells. 

Surrounding the LC is a relatively unstructured sequence of ~100 amino acids that is part of the *N*-terminal sequence of the heavy chain (HC). This “belt”, at least in BoNT/A, seems to sterically protect the binding region for the substrate SNAP-25. It has been suggested that the type A belt acts as a chaperone for the translocation of the LC [24]. The belt has also been conjectured to act as a surrogate or a pseudo-substrate [24]. The function of the belt in BoNT/B is less certain because available 3D structures show that it is shifted away from the substrate binding region [16]. 

The heavy chain (HC, ~100 kDa) has three structurally dissimilar domains. The so-called *C*-fragment is composed of two all-b domains (HC_C_ and HC_N_, ~50 kDa; far left-hand side of Figure 1, red and yellow-orange). Together, the HC domains recognize and bind to nerve terminals in a serotype specific manner using proteins, gangliosides or other macromolecules as receptors [25]. The other domain (HN, ~50 kDa, green) is mainly a-helical (middle portion of Figure 1) and assists in the receptor-mediated internalization and translocation of the toxic moiety [26]. This multistep mechanism of neurotoxin action was first enunciated by Simpson (1980) [27] and has been kinetically modeled [27,28]. It is worth emphasizing that these neurotoxin molecules are not merely proteases. Rather, they are naturally occurring nano-scale machines [11] that bind and enter into cholinergic nerve endings to deliver their enzymatic cargos. The BoNTs are highly selective for their substrates, which regulate vesicle-mediated neurotransmission. 

## 2. Proteolytic Activity

The clostridial neurotoxins are noted for their substrate selectivity and cleavage site specificity. Only BoNT/C1 has been shown to attack two different SNARE proteins, syntaxin1 and SNAP-25 (Table 3; [6,29,30,31,32]). Specificity [33] describes the level of restriction of the cleavage sites at peptide backbone locations in a each substrate. Only TeNT and BoNT/B attack the same substrate (VAMP) at the same peptide bond (Q76-F77, human sequence, UniProtKB accession P63027 [34]). 

The first deduced amino acid sequence of a clostridial neurotoxin was determined for TeNT [35]. From this sequence, TeNT was predicted to be a Zn-dependent protease [36]. This frequently overlooked bioinformatic forecast was based solely on the zincin sequence pattern (HExxH) that is found to be associated with the active-site of the prototypical metalloprotease thermolysin. This prediction was experimentally verified and localized to the LC [18,37,38]. As expected, this protease activity can be blocked by chelators [29,39]. 

The clostridial neurotoxins belong to the gluzincin subgroup of metalloproteases that has the HExxH…E pattern [1,40]. For the BoNT/A sequence (strain Hall / ATCC 3502 / NCTC 13319; UniProtKB accession BXA1_CLOBH), the invariant histidine and glutamate residues (H223, H227, E262) form non-covalent bonds with the zinc ion and the glutamate (E224) is the active site residue. A more detailed version summarizing a 10-residue consensus pattern of the Zn-binding region is described in the Prosite database [20] for neutral zinc metallopeptidases. 

The variability of LC sequence identity percentages ranges from 31-59% when pair-wise alignments are performed with the seven BoNT serotypes and TeNT [41] (Table 1). The substrate locations cleaved by the seven BoNT serotypes are summarized in Table 2 [6]. Representations of the 3D structures of the BoNT holotoxin, LC, C-fragment (HC_C_) and a variety of mutants along with references and other material are located at the BotDB resource (see Table 3).

**Table 1:** Pairwise sequence similarities and identities of the BoNT/A-G and TeNT light chain. % similarity is shown above the diagonal and % identity below the diagonal. Table modified from Lebeda and Olson [41].

% similarities								
	**/A**	**/B**	**/C1**	**/D**	**/E**	**/F**	**/G**	**TeNT**
**/A**	-	52	52	54	54	53	53	52
**/B**	31	-	54	53	58	58	75	68
**/C1**	33	33	-	61	54	56	53	55
**/D**	34	33	56	-	54	55	54	54
**/E**	34	37	35	36	-	72	58	62
**/F**	34	38	35	36	58	-	59	62
**/G**	35	59	35	36	38	40	-	66
**TeNT**	31	50	34	34	44	44	49	-
% identities								

**Table 2:** A summary of the BoNT serotypes, their target SNARE proteins, their intracellular compartments and the cleavage site locations. This table is available at the BotDB website.

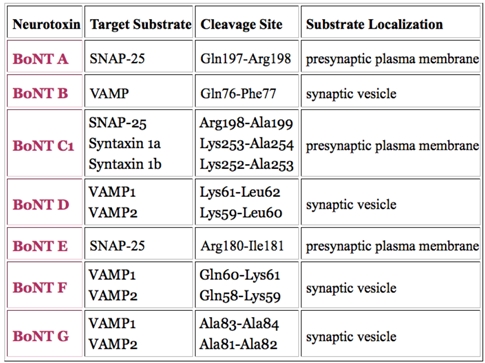

It should be noted that in rats, VAMP1 unlike VAMP2, is not cleaved by BoNT/B because it has VF instead of QF that is located at this cleavage site in the corresponding mouse or human proteins.

**Table 3:** A partial list of 3D structures, which are hyperlinked to other features in the BotDB ^a^.

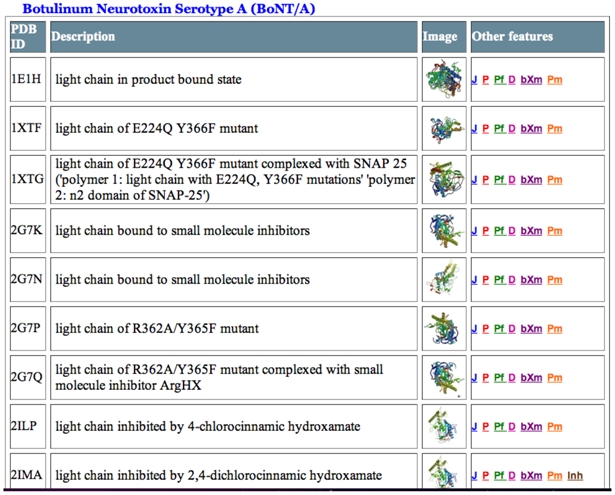

^a ^Abbreviations in the right most column are linked to various sites: J, Jmol graphics program; P, PDB website; Pf, PDB file; D, DSSP Summary; bXm, literature reference from botXminer; Pm, PubMed; Inh, inhibitor.

There is also evidence for the need of relatively long minimal substrates to support cleavage. For example, a 62-mer VAMP fragment for TeNT [42], and a 17-mer SNAP-25 fragment for BoNT/A [43] have been documented. The need of relatively long minimal substrates is consistent with the computational results in which the binding free energy in the model became more favorable as the substrate (a VAMP fragment) bound in a zipper-like manner to form a complex with the LC of BoNT/B [44]. 

One conclusion from these observations is that there is a requirement for the substrate to bind at sites that are distal to the active site. From analysis of multiple sequence alignments, it was noted that substrate selectivity could be provided “by the critical positioning of the substrate by the non-perfectly conserved residues … plus the residues flanking the active-site region“ [45]. From X-ray crystallographic studies by Brunger’s group, the discontinuous binding locations for SNAP-25 are distal to the catalytic site. The term exosites defined these locations on the BoNT/A-LC [46] (PDB ID: 1XTG; Table 3). Substrate specificity was described in terms of an array of exosites. An abundance of binding sites is in accord with the enzyme-substrate interface area of 4,840 Å^2^ for BoNT/A that is about four times larger than the typical protein-protein interface [47].

The structures of the active sites of three neurotoxins (A, B and E) were considered to be virtually identical. From this analysis it was concluded that substrate recognition does not occur at the catalytic machinery [46]. Furthermore, the data showed that substrate binding induced structural changes that probably influenced catalytic activity and that the rational design of specific inhibitors of BoNT/A could benefit from a knowledge of the exosite structures that recognize the substrate [46].

## 3. Enzyme Kinetics

Cell-free assays were initially developed in which the toxin was combined with the appropriate substrate and kinetics of substrate cleavage could be monitored and analyzed [30,41,46,47,48,49]. Initially, samples of holotoxin that were isolated and purified from the organism were used. When recombinant techniques were able to express the LC in *E. coli* or yeast, this toxin fragment replaced the holotoxins in these assays.

**Figure 2:**
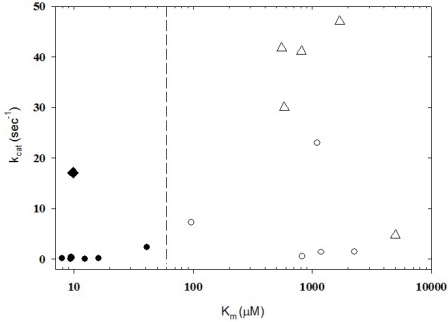
Values of K_m_ and k_cat_ obtained from cell-free assays depend on the forms of the toxic moiety and the substrate molecule used. The LC of BoNT/A (LC-A) and full length SNAP-25 (residues 1-206) are associated with values of K_m_ (closed symbols) that are less than those associated with the LC-A and a 17-mer of SNAP-25 (residues 146-206; open symbols). Larger values for k_cat_ tended to be associated with a 17-mer of SNAP-25 and the holotoxin (open triangles). Open circles: LC-A used with 17-mer SNAP-25 fragment; closed circles: LC-A used with full-length SNAP-25 (1-206) containing His-6 tag. Closed diamond: data associated with the largest k_cat_/K_m_ ratio in this data set (see text). Dashed vertical line: arbitrarily positioned below K_m_ = 100 mM to visually separate high and low values of K_m_. Data collected from [48,49,50] and references therein.

Experimental conditions are critical determinants for the outcomes-a wide range of K_m_ and k_cat_ values have been reported under different cell-free conditions (Figure 2) [48,49,50]. 

In general, experiments with LC-A and SNAP-25 fragments >61 residues or full length substrates produce a range of k_cat_/K_m_ values (10^4^ to 10^6^ s^-1^M^-1^) that is larger compared to the range determined from experiments with LC-A and the 17-mer SNAP-25 fragment (10^2^ to 10^3^ s^-1^M^-1^). Experiments using reduced holotoxin produced a similar quantitative trend, in which the full length substrate was associated with larger values for k_cat_/K_m_ than those observed using the 17-mer fragment. 

As the ratio k_cat_/K_m_ increases, the enzymatic performance usually increases. The term “performance constant” has been suggested for this ratio and is considered to be a more accurate descriptor than the “specificity constant” [51]. The largest ratio in the data set shown in Figure 1 (filled diamond) is 60 s^-1^/16.2 mM or 3.7 × 10^6^ s^-1^M^-1^[52] using the LC-A (1-425) and a 61-mer SNAP-25 fragment. This ratio is 2-3 orders of magnitude below the diffusion limit [53], suggesting that only in a fraction of substrate-enzyme collisions are productive and, therefore, the cleavage reaction appears to be the limiting step. 

This toxin-substrate combination may represent an optimal condition for selecting a standard for testing active-site inhibitors in cell-free assays. Taking into consideration that this ratio has not been measured within the intracellular milieu of presynaptic termini (Section 6), it is currently premature to define standards based on the kinetic values obtained in cell-free systems. Rather a set of different cell-free conditions may be necessary to evaluate the effectiveness of candidate inhibitors (Section 4).

To support the idea that the catalytic step is indeed rate limiting, one can calculate the value of the dissociation reaction rate of the toxin-substrate complex and compare it to the value of k_cat_. Relatively few studies have determined the dissociation constant (K_d_) for the SNAP-25 BoNT/A interaction [50,54]. To achieve this experimentally, mutants were developed to produce a non-cleavable substrate and a value of K_d_ = 2.33 × 10^-7^ M was determined [50]. This value along with the K_m_ and k_cat_ values of the toxin- cleavable substrate reaction, forward (k_1_) and backward (k_-1_) rates for the dissociation reaction can be calculated. Assuming that the following reaction occurs

*k_1_        k_cat_*

E + S ⇌ ES g E + P

*k_-1_*

and that Michaelis-Menten kinetics are obeyed to generate the product (P), the equations for K_m_


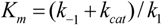
              (1)

and K_d_


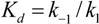
      (2)

can be solved to yield the on rate


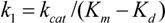
      (3)

and the off rate


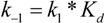
      (4)

of the substrate (S) with respect to the toxin (E). In the experiments using the wild type LC-A and the recombinant SNAP-25 His_6_-tagged substrate, where K_m_ = 9.8 × 10^-6^ M, k_cat_=1.71 × 10^1^ s^-1^, and K_d_ = 2.33 × 10^-7 ^M [50], the calculated values of k_1_ and k_-1_ are 7.7 × 10^12^ s^-1^M^-1^ and 1.8 × 10^6^ s^-1^, respectively. Using equations 3 and 4, the value of k_cat_ is about 10^5^ times slower that k_-1_. Using a LC-A concentration of 100 nM results in a forward rate, k_1_[E] = 7.7 × 10^5^ s^-1^, which is more than 10^4^-times faster than k_cat_. Thus, these calculations support the idea that product formation is the rate-limiting step in reaction 1.

## 4. Inhibitors

In the quest for inhibitors that antagonize the toxic effects produced by the clostridial neurotoxins, well-studied inhibitors of other enzymes, such as thermolysin and angiotensin converting enzyme (ACE), were initially tested. Depending on experimental conditions, the blockade of TeNT activity did not occur with high concentrations of phosphoramidon, thiorphan or trans-epoxysuccinic acid (E64), which inhibits thiol proteases [55]. On the other hand, phosphoramidon, a thermolysin inhibitor, was effective against the TeNT LC [37] or was a weak inhibitor of VAMP cleavage by BoNT/B [56]. Phosphoramidon moderately prolonged the half- times-to-block produced by BoNT/A or B at the NMJ [57]. Captopril, a potent ACE inhibitor, at a relatively high (1.5 mM) concentration only produced a short delay before complete BoNT-induced paralysis in NMJ preparations [57,58]. 

Because of the neurobiology associated with these neurotoxins, blocking their effects may involve more than antagonizing their catalytic activity and, consequently, a variety of additional approaches have been attempted. The toxin domains that take part in the multi-step intoxication reactions for BoNT binding, internalization, translocation and toxicity have been targets of a variety of candidate inhibitors. For example, several naturally occurring lectins containing sialic acid were effective (K_i_ ~ 100 nM) in preventing BoNT/ A, B, C1, D, E, F and TeNT from binding to presynaptic terminals [59]. 

Internalization and translocation of the toxic moiety requires acidic vesicular compartments and metabolic energy. Agents that prevent pH drops that accompany vesicle-mediated endocytosis have been examined. Proton ionophores, nigericin and monensin, which are thought to antagonize the translocation of the LC, produced delays in the production of toxicity in NMJ preparations [58]. Other blockers of vesicular acidification, chloroquine, aminoquinolines [60] and selected quinolines [61] have also been tested. The onset of paralysis in NMJ experiments caused by BoNT/A, B, and C1 were blocked in a concentration-dependent manner with millimolar amounts of ammonium chloride and methylamine hydrochloride. Internalization was delayed and the toxins were susceptible to extracellular antibodies [62]. Uncouplers of oxidative phosphorylation CCCP, FCCP [58] and inhibitors of vesicular H^+^ ATPase [63] have also been examined. Some of these compounds were toxic and, at best, those that were not toxic had low safety margins that prevented them from being considered as therapeutic candidates. In addition, some candidate compounds may transiently antagonize the effects of some serotypes (e.g., BoNT/A). Selected aminopyridines act indirectly by blocking K^+^ channels which prolongs action potential durations and increases Ca^2+^ influx into nerve terminals thereby transiently overcoming the effects of BoNT/A [64,65,66].

With respect to the development of candidate inhibitor compounds, the toxic reaction step involving the proteolysis of one of the SNARE proteins has been the major focus of attention over the past 10 years. A variety of conventional and novel approaches have been used to develop active-site inhibitors and only a few examples will be mentioned here. Extensive *in vivo* testing has been delayed in favor of screening candidate inhibitors using isolated, mouse phrenic nerve-hemidiaphragm preparations [67]. More recently, studies have sought to understand the properties of the reactants and the minimal requirements for proteolysis in cell-free preparations. As mentioned in Section 2, determinations have been made of which substrate fragments can support cleavage by a neurotoxin [32,43,68,69,70,71]. Some of these studies evolved into searches for peptide inhibitors of this reaction including synthetic peptides with proline-rich motifs [72]. Investigations of peptide inhibitors have inspired the development of substrate-based peptidomimetics as novel active-site inhibitors [73].

Low molecular weight organic compounds have been synthesized and screened for inhibitory activity. A crystallographic study of BoNT/B in complex with bis(5-amidino-2-benzimidazolyl) methane revealed that this compound had rearranged the active site, and removed the zinc ion that, presumably, caused the loss of proteolytic activity [74]. Vast quantities of products emerging from combinatorial chemical techniques [75] have been subjected to high throughput screening systems [76]. In contrast, the pharmacophore concept and structure-activity relationships (SAR) for drug discovery have exploited computational chemistry techniques [77]. Computer aided designs have enabled the refinement of a lead compound having an IC_50_ value of 100 mM to one having a K_i_ value of 760 nM [47]. A recent inhibitor designed on SAR principles has been co-crystallized with BoNT/A-LC (PDB ID: 3DSE) and has a K_i_ value of 41 nM [78]. In this case, tight-binding inhibitor kinetics may be more appropriate for analysis than traditional competitive inhibitor models [79]. 

Some of these procedures have led to the development of the first irreversible inhibitor based on a benzylidene cyclopentenedione structure whose mechanism involves covalent modification of the active site [80]. Most recently, the identification of exosites [73] on the LC-A (Section 2) has led to the development of non-competitive blockers of substrate binding at these sites as exemplified by the natural product d-chicoric acid (I1) from *Echinacea* [81]. It will be important to determine how toxic these compounds are in cultured cells and in animal models. From a toxicity perspective, it will be important to understand the findings of Janda’s group [82], in which the two most efficacious compounds *in vivo* showed less activity in cellular assays. Indeed, one of these compounds was cytotoxic at concentrations three orders of magnitude below its effective dose *in vivo*.

**Figure 3:**
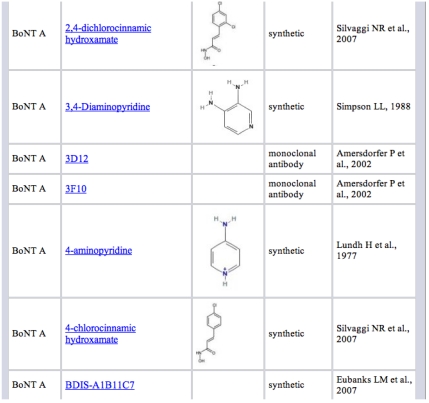
Example list of compounds in the BotDBI that have been evaluated as blockers of BoNT/A action.

Within the BotDB resource, the BotDBI section has information on more than 60 inhibitor candidates including peptides, synthetic and natural compounds, and monoclonal antibodies, along with references. Searches for candidate inhibitors can be done by author name, value of IC_50_, inhibitor site, inhibitor type, value of K_i_, inhibitor name PubMedID BoNT serotype, and inhibitor structure. An example of a list of synthetic compounds in the BotDBI section is shown in Figure 3.

**Figure 4:**
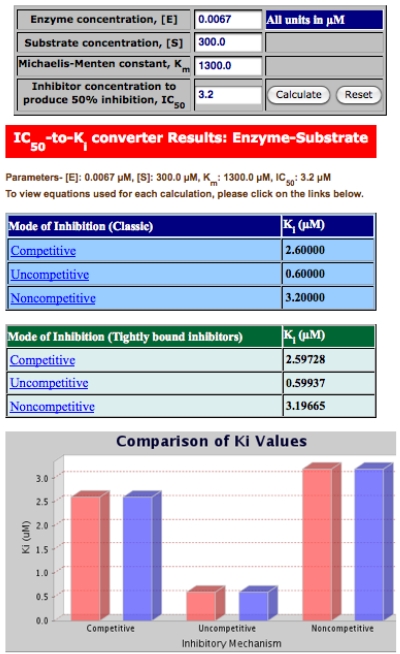
Output of the IC_50_-to-K_i_ converter tool at the BotDB website that shows the different possible results based on whether the inhibitor mechanism is competitive, uncompetitive or noncompetitive.

In another part of the BotDB resource is an on-line, user friendly tool that converts IC_50_ to K_i_ values depending on whether the inhibitory mechanism is competitive, uncompetitive, or noncompetitive (Figure 4) [79]. The upper portion of this output lists the parameter values (in mM units) that need to be entered by the end user, *i.e.*, enzyme (toxin) concentration, substrate concentration, K_m_ and IC_50_. The middle panels display the results from the K_i_ calculations assuming that three different mechanisms follow either the classic (Michaelis-Menten) scheme or are associated with a tightly bound inhibitor. The bottom panel has color-coded histograms for classic (red bars) and for tightly bound (blue bar) inhibitors from six different calculations for the K_i_ values. This display allows the user to visualize and readily compare the results. 

## 5. Activators

Schmidt and Bostian [83] described the stimulating effects of bovine serum albumen (BSA) and other serum albumins that were kinetically analyzed by following the BoNT/A-mediated cleavage of the 17-mer SNAP-25 fragment by BoNT/A. Addition of 1 mg/ml BSA, produced a 10-fold increase in the value of k_cat_ and a three-fold decrease in K_m_ leading to an increase of 30-fold for the performance constant (k_cat_/K_m_, Section 3). This enhancing effect of BSA has been noted with other enzymes [84].

Beyond an apparent non-specific stabilizing effect that may underlie the action of BSA, some low-molecular weight synthetic ligands having a 2-acylguanidyl-5-phenyl thiophene scaffold have been reported to activate the enzymatic effects of BoNT/A under cell-free conditions [85]. In that study, the value of K_m_ was reduced by the ligands while the value of k_cat_ was essentially unaffected. Importantly, this activation was determined not to be a detergent-like effect. The results were so striking that these authors described their compounds as being super-activators. 

One implication of these findings may affect the use of these toxins as pharmaceutical treatments for a variety of conditions and disorders. A better therapeutic strategy may be created if these activating compounds were part of the toxin formulation. Ideally, less toxin would be needed to produce an optimal level of therapeutic benefit, thus reducing the incidence of toxic side effects and causing or development of resistance to treatment. 

At higher concentrations, some ligands may exhibit both stimulatory and inhibitory effects. The theoretical basis for this dual mechanism for a single ligand have been kinetically analyzed in detail by others, e.g., [86,87]. Such a concentration-dependence is predicted to be observed in cell-free systems using a wider range of inhibitor and substrate concentrations than have thus far been examined.

## 6. Future Research

The long-lasting effects of botulism are well known [12,14,88] with patients still presenting some of the toxic signs and symptoms a year after onset. In one extreme case, the effects due to type A toxin poisoning persisted for over five years [89]. It is clinically relevant to understand the underlying mechanism of persistence. As mentioned in Section 5, the presence of an additive that prolongs the persistence of BoNT-induced effects could be exploited by caregivers to provide longer lasting therapeutic effects with a reduced number of injection sessions along with fewer and less severe adverse reactions for the patient.

Two hypotheses have been advanced. The long lasting activity of the type A toxin was hypothesized to be based on the longevity of the LC within the nerve terminal [90,91] or to a slower turnover of the truncated SNAP-25 generated by BoNT/A-mediated proteolysis. Presently, there is no direct evidence for either the BoNT LC or for the SNAP-25 cleavage products to have a long half life. Moreover, the proposed long-lived activity of the BoNT/A LC is apparently not due to an extraordinary thermostability compared to that of thermolysin. This is because the estimated activation energy for unfolding, E_a_, is ~ 9 kJ/mol for the reduced form and 22 kJ/mol for the unreduced form of BoNT [92]. These values are low in comparison with the E_a_ for inactivation of the more temperature stable thermolysin which is ~149 kJ/mol [93]. 

Dolly and colleagues challenged the view that BoNT/A activity was extraordinarily stable. These researchers hypothesized that the protracted time course of the toxic effects was due to the truncated SNAP-25 that results from BoNT/A-mediated cleavage of the last nine C-terminal residues [94,95,96]. Evidence was presented in which one of the cleavage fragments formed a long-lasting but incompetent SNARE complex with syntaxin and VAMP. Normally the SNARE complex dissociates into the three monomers in conjunction with a-SNAP [a-soluble *N*-ethylmaleimide-sensitive fusion protein (NSF) attachment protein] and NSF [97,98].

An issue that may be related to the persistence phenomenon is the differential localization of the LCs for BoNT/A and E. A di-leucine motif is present in the LC of BoNT/A, but does not exist in the LC of BoNT/E. It is believed that this motif functions as a sorting signal to keep the LC-A associated with the cytoplasmic surface of the nerve terminal, while the LC-E is removed from this site and presumably metabolized into an inactive form [99].

Focusing on the LC stability portion of this hypothesis, mechanisms unique to the intracellular environment may lead to persistence of LC-A within nerve terminals that may not occur in cell-free systems. For example, persistence of proteolytic activity could be due to post-translational modifications (PTMs) of LC-A or as a consequence of interactions of LC-A with molecules that serve as molecular chaperones. Like other enzymes, BoNT is regulated through post-translational modifications such as the nicking process that creates the LC and HC (Section 1) along with the reduction of the interchain disulfide bond that is essential to the activation of BoNT or TeNT [29,38]. What is not known to any great extent are other possible covalent modifications in which low molecular weight groups are added. The existence of phosphorylation of tyrosines of the type A, B, E and TeNT LCs [100], which may enhance its thermal stability while retaining its catalytic properties has been reported but is of uncertain functional significance within neurons [101]. Within the environment of the nerve terminal, rate constants (Section 3) may be affected by molecular crowding or confinement [102]. Protein structural stability and enzymatic efficiency could be enhanced by endogenous substances that are usurped by the toxin to act as molecular chaperones. Such concepts provide a physical basis for long-lived enzyme activity when considering persistence of BoNT/A and perhaps other serotypes.

Because the persistence hypotheses considered above are not mutually exclusive, both could be merged into a single framework to account for long-term paralysis. Future research is required to advance our understanding of this intriguing phenomenon and the related problem of developing activator additives to improve the BoNT therapeutic effects of the BoNTs. In addition, continued improvements are needed in developing inhibitors to ameliorate BoNT toxicity. These would be used for treatment of overdose following clinical use, for treatment of accidental intoxication in the human and animal populations and most importantly for treatment of casualties after a bioterrorist attack.
